# B-cell acute lymphoblastic leukemia in an elderly man with plasma cell myeloma and long-term exposure to thalidomide and lenalidomide: a case report and literature review

**DOI:** 10.1186/s12885-019-6286-9

**Published:** 2019-11-27

**Authors:** Ryan B. Sinit, Dick G. Hwang, Prakash Vishnu, Jess F. Peterson, David M. Aboulafia

**Affiliations:** 1 Floyd and Delores Jones Cancer Institute, Virginia Mason Medical Center, 1100 Ninth Avenue (C2-HEM), Seattle, WA 98101 USA; 2 Department of Pathology, Virginia Mason Medical Center, Seattle, WA USA; 3 Department of Medical Oncology, Mayo Clinic, Jacksonville, FL USA; 4 Division of Laboratory Genetics and Genomics, Department of Laboratory Medicine and Pathology, Mayo Clinic, Rochester, MN USA; 5 Division of Hematology, University of Washington School of Medicine, Seattle, WA USA

**Keywords:** Myeloma, Lymphoblastic leukemia, Lenalidomide, Thalidomide, Therapy-related cancer

## Abstract

**Background:**

The advent of the immunomodulatory imide drugs (IMiDs) lenalidomide and thalidomide for the treatment of patients with plasma cell myeloma (PCM), has contributed to more than a doubling of the overall survival of these individuals. As a result, PCM patients join survivors of other malignancies such as breast and prostate cancer with a relatively new clinical problem – second primary malignancies (SPMs) – many of which are a result of the treatment of the initial cancer. PCM patients have a statistically significant increased risk for acute myeloid leukemia (AML) and Kaposi sarcoma. IMiD treatment has also been associated with an increased risk of myelodysplastic syndrome (MDS), AML, and squamous cell carcinoma of the skin. However, within these overlapping groups, acute lymphoblastic leukemia (ALL) is much less common.

**Case presentation:**

Herein, we describe an elderly man with PCM and a 14-year cumulative history of IMiD therapy who developed persistent pancytopenia and was diagnosed with B-cell acute lymphoblastic leukemia (B-ALL). He joins a group of 17 other patients documented in the literature who have followed a similar sequence of events starting with worsening cytopenias while on IMiD maintenance for PCM. These PCM patients were diagnosed with B-ALL after a median time of 36 months after starting IMiD therapy and at a median age of 61.5 years old.

**Conclusions:**

PCM patients with subsequent B-ALL have a poorer prognosis than their de novo B-ALL counterparts, however, the very low prevalence rate of subsequent B-ALL and high efficacy of IMiD maintenance therapy in PCM should not alter physicians’ current practice. Instead, there should be a low threshold for bone marrow biopsy for unexplained cytopenias.

## Background

Plasma cell myeloma (PCM), previously referred to as multiple myeloma, is a neoplasm of plasma cells and is the second most common hematologic malignancy [1–3]. Virtually all cases of PCM are preceded by monoclonal gammopathy of undetermined significance (MGUS). Progression from MGUS to smoldering (asymptomatic) PCM arises from a random second event e.g., additional genetic changes from chronic antigenic stimulation or exposure to toxins, cell cycle dysregulation, or changes in the bone marrow microenvironment [4, 5]. Active (symptomatic) PCM is related to the expansion and infiltration of plasma cells within the bone marrow and other end-organs, resulting in significant clinical events including bone demineralization, pathologic compression fractures, cytopenias, hypercalcemia, and renal dysfunction.

Smoldering PCM is not always treated but does require close clinical follow-up [6]. Symptomatic PCM is typically treated with induction therapy which may include combinations of corticosteroids, proteasome inhibitors, immunomodulatory imide drugs (IMiDs), DNA alkylators and, less commonly, anthracyclines and monoclonal antibodies. Eligible patients often undergo consolidation therapy with high-dose melphalan and autologous hematopoietic stem cell transplant (AHSCT) and subsequent maintenance therapy with an IMiD or a proteasome inhibitor [1, 7–9].

The advent of new therapeutic agents has dramatically improved survival for patients, particularly those who are younger than 65 years of age at diagnosis [10, 11]. Consequently, patients with PCM, like survivors of other malignancies, most notably, breast cancer, prostate cancer, Hodgkin’s lymphoma (HL), and non-Hodgkin’s lymphomas (NHLs), are confronted with a relatively new clinical problem—second primary malignancies (SPMs) which include both presumed treatment-related cancers and de novo second cancers. Analysis of the National Cancer Institute’s (NCI) Surveillance, Epidemiology, and End Results (SEER) database of all registered cancer patients between the years 1973 and 2000 showed a 14% increased risk of a second malignancy for these patients compared to the general population [2]. Since the increased overall survival of PCM patients is a relatively new phenomenon, the incidence of SPMs for these patients is still relatively lower than all the other cancers, reported to be 4.5% – which is lower than both HL and NHLs (both occurring at an incidence of 6.4%). When stratified by cancer type and compared to the expected rate of malignancies in the general population, PCM patients had a statistically significant elevated risk for hematopoietic malignancies and Kaposi sarcoma. Myeloid leukemias are the most common hematologic malignancies with acute myeloid leukemia (AML) constituting 80% of all cases of leukemia following PCM [2].

As survival of PCM patients continues to increase, so too will the number of SPMs attributable to myeloma treatments. Though likely a multifactorial process, studies have associated the use of IMiDs with myelodysplastic syndrome (MDS), AML, and squamous cell carcinoma of the skin [9, 11–19]. Secondary B-cell acute lymphoblastic leukemia (B-ALL) has been reported only rarely.

Herein, we describe the case of evolving cytopenias in an elderly man with a 14-year history of IMiD treatment with recurrent skin cancers and a more recent finding of concomitant progressive PCM and B-ALL.

## Case presentation

A 67-year-old Caucasian man had been in generally good health; his medical history was significant only for a resected insulinoma. In late 2003, he presented to medical attention with altered mental status and was diagnosed with pneumococcal pneumonia and bacterial meningitis. He had an elevated total protein of 12.6 g/dL (normal, < 8.0 g/dL) and albumin of 3.3 g/dL (normal, 3.4–4.8 g/dL). Serum protein electrophoresis (SPEP) showed a monoclonal (M) IgG kappa spike of 7.0 g/dL. Metastatic bone survey revealed multiple lytic lesions principally involving the cervical spine and right humerus. A bone marrow biopsy showed > 50% monotypic plasma cells and normal karyotype.

**Fig. 1:**
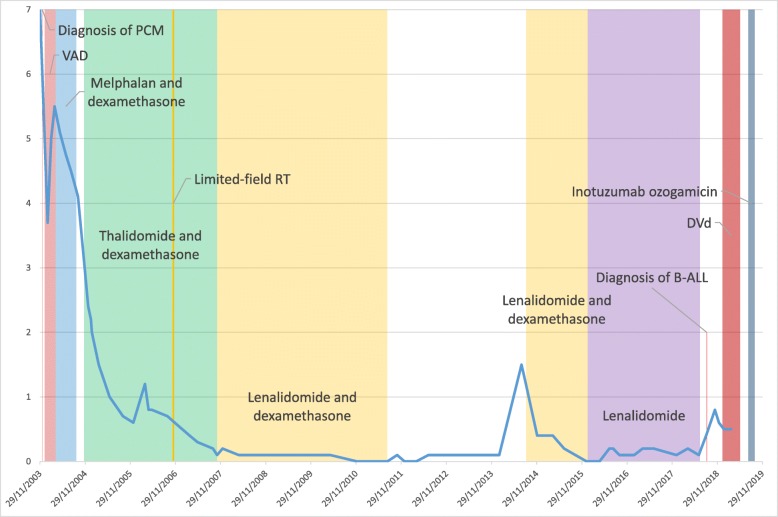
Treatment of plasma cell myeloma and corresponding monoclonal protein concentration by serum electrophoresis

He began treatment for PCM in December 2003 with a regimen consisting of vincristine, doxorubicin, and dexamethasone (VAD) (Fig. 1). Despite an initial positive response, after three cycles, he had an abrupt increase in M protein. The treatment was therefore changed to oral melphalan and prednisone which he continued for 5 months. M protein declined significantly, but due to pancytopenia, the melphalan regimen was replaced by thalidomide and dexamethasone. He continued to take thalidomide in conjunction with dexamethasone for 36 months. Treatment was held for a month in 2006 after he sustained bilateral pathologic humeral fractures for which he received 3000 cGy of radiation therapy divided equally over 10 fractions.

While on thalidomide and dexamethasone, his PCM was well controlled, but worsening peripheral neuropathy prompted a switch to lenalidomide in November of 2007. With this change, PCM remained well controlled for the next 4 years. However, in mid-2011, lenalidomide was discontinued after he was diagnosed with a stage 1B melanoma of the right forearm which was treated with a curative wide local resection. In the 3 years prior to the melanoma diagnosis, he had also received local treatments for squamous cell cancer of the scalp and basal cell cancer over the zygomatic arch.

For 3 years after discontinuation of lenalidomide, the patient’s PCM remained quiescent with an undetectable M protein, but during a routine follow-up evaluation in September 2014, the serum M protein increased to 1.5 g/dL, thus lenalidomide and dexamethasone were reintroduced. After 15 months, dexamethasone was discontinued to minimize the toxicity of chronic steroid therapy and he continued lenalidomide monotherapy. Over the next 2 years, he maintained an M protein of ≤0.2 g/dL.

**Fig. 2:**
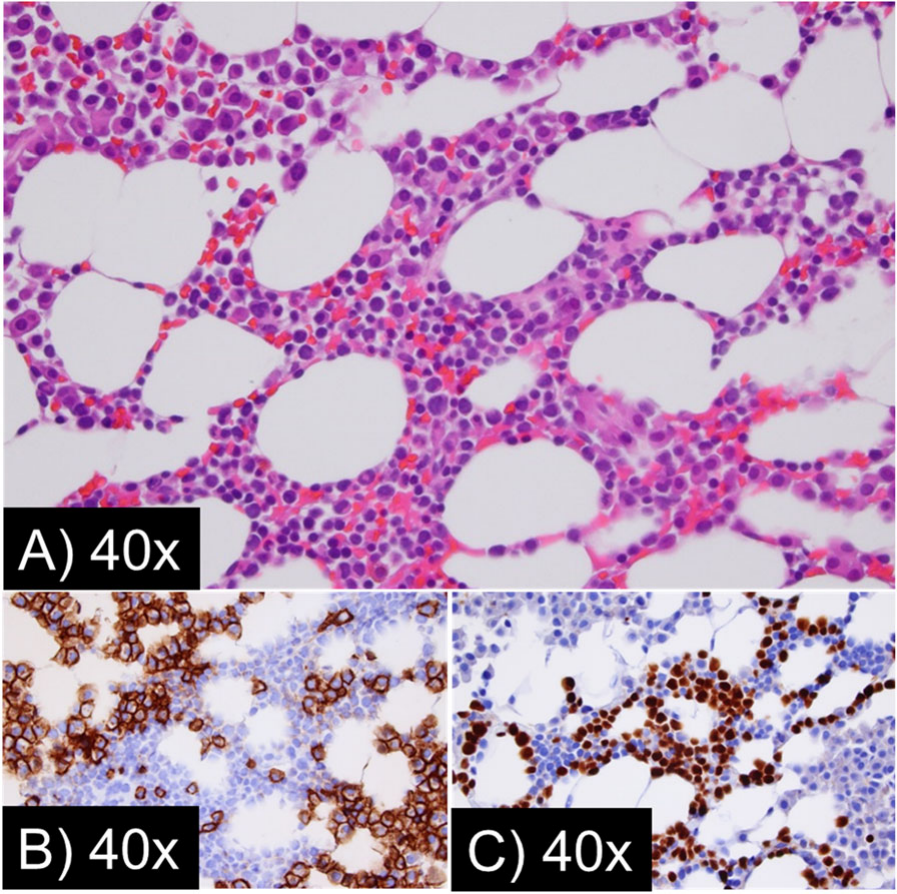
Left iliac crest bone marrow aspirate from September of 2018 showing two concurrent processes: B-cell acute lymphoblastic leukemia and plasma cell myeloma. **a** Hematoxylin and eosin (H&E) stain. **b** CD138 stain highlighting the neoplastic plasma cells of plasma cell myeloma. **c** Terminal deoxynucleotidyl transferase (TdT) stain highlighting the lymphoblasts of B-cell acute lymphoblastic leukemia. The three panels show the same field of view, and the two processes can also be seen on H&E as two morphologically distinct populations

**Fig. 3:**
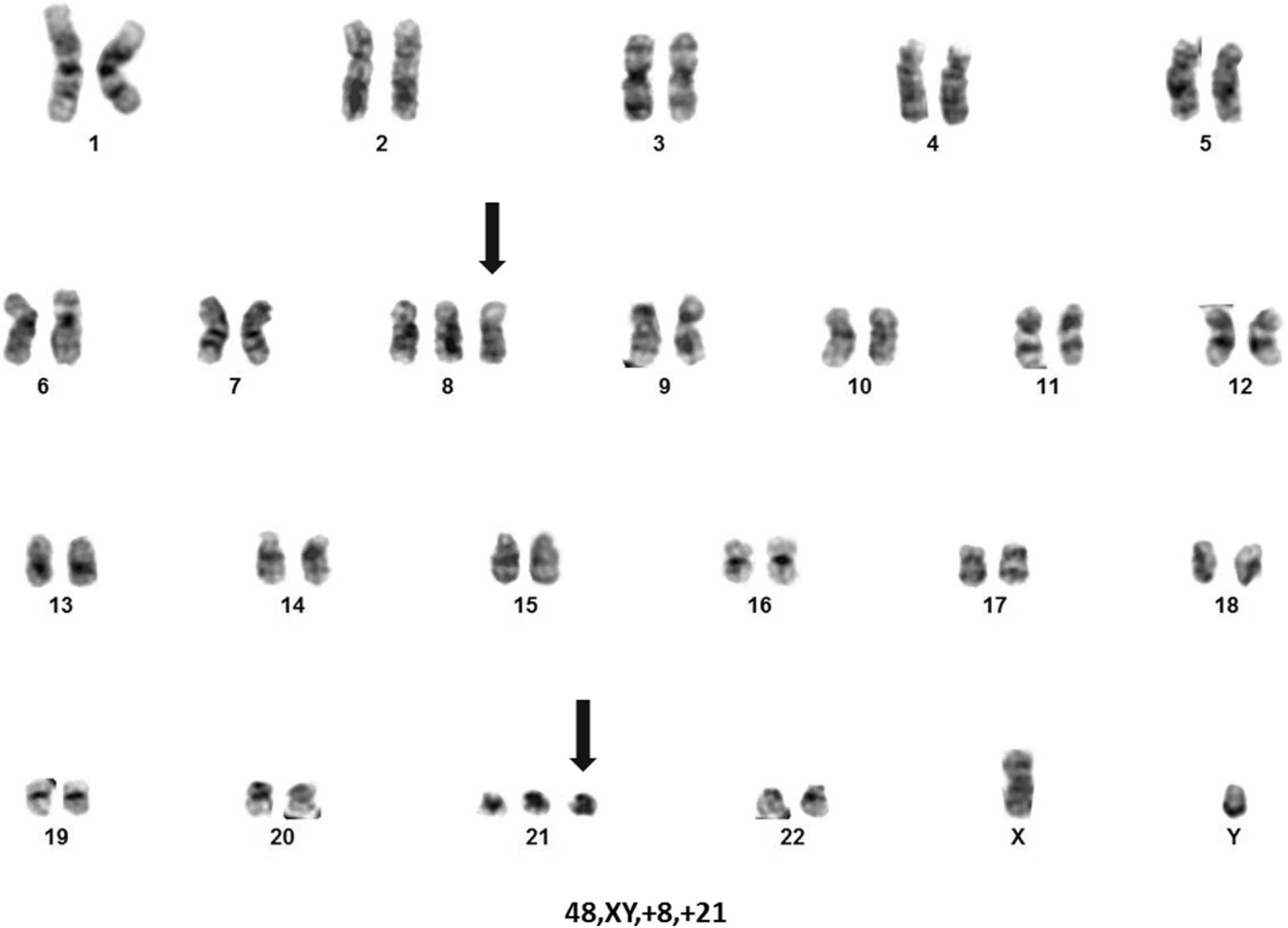
Representative karyogram obtained from the patient’s bone marrow aspirate from September of 2018 showing trisomies 8 and 21. Fluorescent in situ hybridization studies also revealed intact copies of *MYC* (8q24) and *RUNX1* (21q22) which is consistent with conventional chromosome studies

In 2018, at age 82, during a routine follow-up evaluation, his white blood cell (WBC) count was 0.8 × 10^9^ cells/L (normal, 3.5–11.0 × 10^9^ cells/L), hematocrit 32% (normal, 39–50%), platelet count of 89 × 10^9^ cells/L (normal, 150–400 × 10^9^ cells/L), and a M protein concentration of 0.4 g/dL. Lenalidomide was held and when cytopenias did not improve, a posterior iliac crest bone marrow aspirate and core biopsy were obtained. Pathology findings of the marrow core biopsy showed monotypic plasma cells comprising 20% of the marrow cellularity consistent with preexisting PCM; however, 60% of the marrow cellularity was comprised by lymphoblasts with a morphology and immunophenotype consistent with B-ALL (Fig. 2). Qualitative polymerase chain reaction was negative for the Philadelphia chromosome (BCR-ABL1 fusion). Cytogenetic analysis revealed trisomies 8 and 21 in 2 of 20 metaphases and a near-tetraploid population of interphase cells including trisomies 8 and 21 (Fig. 3). Fluorescence in situ hybridization (FISH) studies for common chromosome abnormalities associated with B-ALL on interphase cells revealed a near tetraploid clone in 25% of nuclei. In addition, approximately 13% of nuclei had 3 intact copies of *MYC* (8q24) and *RUNX1* (21q22), consistent with the trisomy 8 and trisomy 21 anomalies observed in the chromosome studies. FISH probe sets on plasma cells using immunoglobulin staining demonstrated a plasma cell clone with deletion of the *TP53* gene region, trisomies 3, 7, 11, and trisomies/tetrasomies 9 and 15.

For 6 months, the patient remained asymptomatic but with evolving red cell transfusion dependence, increasing WBC to 30 × 10^9^ cells/μL, 12% blasts in peripheral blood, increasing M protein, and new bone lesions, he once again began palliative chemotherapy consisting of liposomal doxorubicin, vincristine, and dexamethasone (DVd) in addition to Zoledronic acid. His treatment was complicated by staphylococcal sepsis and severe and protracted pancytopenia. After recuperating, he received a second, but reduced dose of DVd which provided a good response with a resolution of blasts for a brief period of time.

At time of relapse, he began inotuzumab ozogamicin (InO), a CD22 monoclonal antibody antagonist conjugated to calicheamicin. Since initiation of InO, there have been no circulating blasts by flow cytometric analysis 15 months from diagnosis of B-ALL. His clinical course has been complicated by low blood counts. M-protein studies throughout his B-ALL treatment have remained stable at around 0.5 g/dL.

## Discussion

PCM accounts for 1.2% of all cancers and 2% of all cancer deaths in the United States [20]. The five-year relative survival rate for PCM has increased more than two-fold since the NCI’s SEER program started tracking this information; the five-year survival rate was 24.6% for those diagnosed between 1975 and 1977 compared to 52.4% of those diagnosed between 2008 and 2014. The biggest improvement in five-year survival was from 34.6 to 42.5% between 1999 and 2001 and 2002–2004 corresponding to when thalidomide was starting to be used off-label for PCM before it was approved by the Food and Drug Administration in 2006 along with lenalidomide [20–23].

SPMs are becoming increasingly common in cancer survivors due in part to the higher survival rates of some cancers and in part to the improved treatments used to cure them [14, 24]. Most SPMs (80%) arise in separate or independent organ systems from their primary cancers. Though not directly assessed in the SEER SPM analysis, there was an elevated risk of acute leukemias following the treatment of several cancers [2]. The etiology of any SPM is multifactorial, however increasing numbers of reviews have identified a more causal association between the treatment of primary malignancy including chemotherapy and immunosuppression, and the onset of secondary cancers [25].

The first report describing SPMs following PCM was in 1979 wherein 14 of the 364 patients (3.8%) had developed acute leukemia following treatment of PCM with various melphalan-containing regimens [2, 13]. Long-term studies of IMiD maintenance therapy in PCM patients have revealed a higher number of SPMs compared to patients who did not receive long-term lenalidomide. Combining data from two studies published in 2012, 48 of 537 patients (8.9%) who were treated with lenalidomide developed a SPM compared to 20 of 523 (3.8%) who received placebo maintenance [9, 26]. Several meta-analyses have also identified the significant contribution of IMiD and alkylating agent-based therapy to the risk of SPMs [18, 27–33]. In an evaluation of PCM patients who received a variety of different chemotherapy regimens, risk factors of developing MDS and subsequent AML included the use of IMiDs, older age, male gender, and a low reinfusion dose of CD34+ cells following first AHSCT [16]. A more recent analysis performed by the International Myeloma Working Group (IMWG) in 2017 also found a significantly increased incidence of SPMs following the use of lenalidomide and melphalan [12].

The mechanisms by which lenalidomide and other IMiDs contribute to SPMs are unclear. The IMiDs exert their antineoplastic effect through mechanisms such as direct cytotoxicity and indirect effects on tumor immunity [34]. Lenalidomide and thalidomide can also reactivate the lytic cycle of the Epstein-Barr virus in resting memory B-cells and lead to various lymphoproliferative disorders [35].

The vast majority of hematologic SPMs following PCM have been myeloid in nature –cases of B-ALL have been reported far more infrequently and it is unknown if the risk factors for ALL are different from those contributing to myeloid leukemias [17, 18]. To identify ALL cases following PCM, we used PubMed and the search terms “Myeloma and acute lymphocytic leukemia,” “Leukemia following plasma cell myeloma,” “Second primary malignancies and myeloma,” and “IMiD-associated cancers.” We identified an additional 17 patients in the English and non-English-language literature who were diagnosed with B-ALL following PCM (Table 1). An additional two patients had B-ALL after treatment for PCM with high-dose melphalan and AHSCT [36, 37].

**Table 1:** Summary of the cases and pathologic features of B-cell acute lymphoblastic lymphoma following immunomodulatory imide treatment of plasma cell myeloma

Patient^Ref^, Year^b^ (age^a^, sex)	PCM tx (mo on tx)	B-ALL Presentation	B-ALL Molecular Profile	B-ALL Cytogenetics	B-ALL treatment	Response/Duration
Index pt., 2019 (82, Male)	VAD (3) Mel/dex (5) Thal/dex (36) Len/dex (15) Len (31)	Increasing M protein (0.4 g/dL) HCT (32%) WBC count (0.8 × 10^9^ cells/L) Platelet count (89 × 10^9^ cells/L)	Marrow: 60% lymphoblasts +: CD34, 138, TdT Mixed: CD10, 45, HLA-DR -: CD20, 38, Ph	Trisomy 8 + 21	DVd	PR (15+ mo)
Pt 1 [18], 2018 (53, Female)	RT VD (NS) AHSCT Len (72)	Leukopenia Thrombocytopenia	Marrow: 50% lymphoblasts +: CD19, 34, 38, 79a, PAX5, TdT	Trisomy 8 + 10 + 21 Copy gain MYC, IgH Monosomy 2	CALGB 8811 POMP maintenance	CR (12+ mo)
Pt 2 [18], 2018 (69, Female)	Bortezo/len/dex (NS) ASHCT Len (23)	M protein spike (0.5 g/dL)	Marrow: 25% lymphoblasts +: CD19, 22, 34 -: Ph	46, XX, t(7;19)	Hyper-CVAD POMP maintenance	CR (36+ mo)
Pt 3 [29], 2017 (59, Male)	Bortezo/len/dex (5) AHSCT Mel (NS) Len (30)	Fatigue Neutropenia Thrombocytopenia	Marrow: 20% lymphoblasts +: CD10, 19 Mixed: CD20, 79a, PAX5, TdT -: Ph	Deletion 20q	Linker regimen Flu/mel/allo-SCT	CR (12+ mo)
Pt 4 [29], 2017 (34, Male)	Dex (1) Thal/dex (36) Len (36)	Pancytopenia Circulating lymphoblasts	Marrow: near 100% lymphoblasts +: CD10, 19, 22, 34, 38, 79a	Chr 14 rearrangement	CALGB 8811	Died during induction
Pt 5 [29], 2017 (53, Male)	RT Bortezo/len/dex (12) Mel/AHSCT Len (84)	Leukopenia Neutropenia	Marrow: 20–30% lymphoblasts +: CD19, 34, 79a, PAX5, TdT -: Ph	Tetraploidy	Linker regimen AHSCT/BEAM	CR (12+ mo)
Pt 6 [28], 2016 (66, Male)	VD (2) Len (2) VADM (2) Thal (31)	WBC count (3.05 × 10^9^ cells/L) No monoclonal band on SPEP	Marrow: 62% lymphoblasts +: CD19, 22, 34, 45, TdT Mixed: CD79a	Trisomy 4	CHOP	Died during induction
Pt 7 [31], 2016 (65, Female)	VADM (2) Len (2) VD (2) Thal (33)	Dizziness	Marrow: 68% lymphoblasts +: CD19, 22, 34, 45, 79a, HLA-DR -: CD20, 54, 38, 138, Ph	NS	No treatment	Died
Pt 8 [31], 2016 (63, Male)	PAD (4) AHSCT RT VTD (1) Thal (32)	Fatigue Platelet count (33 × 10^9^ cells/L) No monoclonal band on SPEP	Marrow: 84% lymphoblasts +: CD10, 34, HLA-DR -: CD20, 38, 138	NS	Lost to follow-up after diagnosis	
Pt 9 [31], 2016 (33, Female)	DVd (1) VD (4) AHSCT Thal (73)	Cough Fatigue Hgb (10.9 g/dL) No monoclonal band on SPEP	Marrow: 84% lymphoblasts +: CD10, 19, 22, 34, 79a, HLA-DR -: CD20, 25	NS	No treatment	Lost to follow-up
Pt 10 [27], 2013 (62, Female)	VD (4) High-dose dex (2) VBMCP/VBAD (5) Len/dex (3) AHSCT Len (20)	Fatigue Pancytopenia	+: Ph, CD10, 19, 20, 22, 79a, TdT -: CD34	46, XX	NS	Died during induction
Pt 11 [33], 2013 (72, Male)	VAD (4) RT Len/dex (36)	Pancytopenia No evidence of active MM	Marrow: 66% lymphoblasts +: CD10, 19, 20, 79a, TdT -: CD34, 38	NS	Hyper-CVAD POMP maintenance	CR (12 mo) then relapse
Pts 12–14 [36], 2012 (NS, NS)	AHSCT Len (NS)	NS	NS	NS	NS	NS
Pt 15 [26], 2012 (NS, NS)	AHSCT Len (NS)	NS	NS	NS	NS	NS
Pt 16 [30], 2012 (61, Female)	VCMP (7) VMAP (4) Thal (29)	Pancytopenia No monoclonal band on SPEP	Marrow: 93% lymphoblasts +: CD19, 38 -: CD10, 20, 23, 138	46, XX	CALGB 9911 Relapse: VP	CR (10 mo) then relapse Relapse: CR (12+ mo)
Pt 17 [32], 2012 (56, Female)	VCMP (7) Mel/thal/dex (16) Thal (53)	Edema Dyspnea WBC count (3.077 × 10^9^ cells/L) Hgb (8.6 g/dL) Platelet count (80 × 10^9^ cells/L)	Marrow: 37.8% lymphoblasts +: CD10, 19, 20, 22, HLA-DR -: Ph	46, XX	Steroids	Died

*Abbreviations: AHSCT* autologous hematopoietic stem cell transplant, *ALL* acute lymphoblastic leukemia, *allo-SCT* allogenic stem cell transplant, *BEAM* carmustine, etoposide, cytarabine, and melphalan, *bortezo* bortezomib, *CALGB 8811* Cancer and Leukemia Group B Protocol 8811: daunorubicin, vincristine, prednisone, pegasparagase, and cyclophosphamide, *CALGB 9111* Cancer and Leukemia Group B Protocol 9111: cyclophosphamide, daunorubicin, vincristine, prednisone, and L-asparaginase with granulocyte-colony stimulating factor, *CHOP* cyclophosphamide, doxorubicin hydrochloride, vincristine sulfate, and prednisone, *Chr* chromosome, *CR* complete response, *dex* dexamethasone, *DVd* liposomal doxorubicin, vincristine, and dexamethasone, *flu* fludarabine, *HCT* hematocrit, *Hgb* hemoglobin, *hyper-CVAD* hyperfractionated cyclophosphamide, vincristine, doxorubicin hydrochloride, dexamethasone, and alternating high-dose methotrexate and cytarabine, *InO* inotuzumab ozogamicin, *len* lenalidomide, *Linker regimen* daunorubicin, vincristine, prednisone, and pegaspargase, *M* monoclonal, *mel* melphalan, *mo* months, *NS* not stated, *PAD* bortezomib, dexamethasone, and doxorubicin, *PCM* plasma cell myeloma, *PD* progressive disease, *Ph* Philadelphia chromosome (BCR-ABL1 fusion), *POMP* 6-mercaptopurine, vincristine, methotrexate, prednisone, *PR* partial response, *pt* patient, *Ref* reference, *RT* radiation therapy, *SPEP* serum protein electrophoresis, *TdT* terminal deoxynucleotidyl transferase, *thal* thalidomide, *tx* treatment, *VAD* vincristine, doxorubicin, and dexamethasone, *VADM* vincristine, pirarubicin/doxorubicin, dexamethasone, and melphalan, *VBAD* carmustine, doxorubicin, and dexamethasone, *VBMCP* vincristine, carmustine, melphalan, cyclophosphamide, and prednisone, *VCMP* vincristine, cyclophosphamide, melphalan, and prednisone, *VD* bortezomib and dexamethasone, *VMAP* vincristine, melphalan, doxorubicin, and prednisone, *VP* vincristine and prednisone, *VTD* bortezomib, dexamethasone, and thalidomide, *WBC* white blood cell

^a^ age at diagnosis

^b^ year reported

Among IMiD-treated PCM patients with B-ALL, our patient is the oldest (median, 61.5 years; range, 33–82). With a cumulative exposure of 82 months, our patient is also among those with the most extensive duration of IMiD exposure (median, 35.5 months; range, 23–96), and longest latency period between starting IMiD therapy and developing ALL (median, 36 months; range, 20–179). Like the majority (86%) of cases, our patient presented with worsening pancytopenia prompting a bone marrow biopsy which showed progressive PCM and a new B-ALL. Studies of the plasma cells in the aspirate were notable for a *TP53* deletion and trisomies 3, 7, and 11, and trisomies/tetrasomies 9 and 15. *TP53* deletion is associated with an unfavorable PCM prognosis, regardless of other abnormalities detected [38]. On interphase blast cells, FISH demonstrated a near tetraploid clone and trisomies 8 and 21; however, the prognostic significance of these findings in B-ALL is unclear [39]. Our patient is also significant in that he has had other IMiD therapy-related malignancies; basal and squamous cell carcinoma of the skin and melanoma [19].

Treatment modalities for B-ALLs following PCM have varied significantly and include standard B-ALL cytotoxic chemotherapy regimens, corticosteroid monotherapy, or forgoing therapy completely (Table 1). In our patient, advanced age combined with evolving cytopenias precluded traditional ‘induction-type’ cytotoxic treatment regimens for B-ALL. We elected to use DVd as this regimen has overlapping benefits for PCM and B-ALL. In addition, this therapy can be delivered in the clinic without need for protracted hospitalizations. Zoledronic acid was added to minimize the risk of skeletal fractures. At time of progression, he began InO. InO was approved in 2017 and quickly integrated into the NCCN guidelines as an option for patients with relapsed/refractory ALL after showing significant improvements in progression-free survival and overall survival when compared to standard-of-care therapies [40, 41].

Outcomes of PCM patients with hematologic SPMs are worse than those of their de novo counterparts [24]. In an analysis of 27,000 Swedish patients with PCM and MDS or AML, survival outcomes were poorer when compared to matched patients with de novo MDS or AML – a 1.7-fold increased mortality risk was identified in the former cohort when compared to the latter. In addition, the median survival was only 2.4 months and the one-year survival rate was 16% for the former group [15]. Compared to the patients identified in our literature review, our patient was the only one to have poor cytogenetic prognostic features although many of these patients were identified at a time when FISH was not routinely performed and many of the cytogenetic aberrations identified remain of uncertain significance.

Despite the increased risk and poor prognosis of PCM patients with certain SPMs, as of 2017, the IMWG does not recommend alterations to therapeutic decision-making because the overall risk of developing a SPM is low and the therapeutic benefit of IMiD maintenance therapy significantly outweighs the risk of SPMs [12, 26, 42]. They instead recommend a low threshold for bone marrow analysis for patients with unexplained cytopenias following lenalidomide therapy withdrawal [12]. Additional research is needed to identify the weight of each risk factor, with a careful evaluation of the role of IMiDs leading to SPMs.

## Conclusion

In conclusion, B-ALLs are uncommon following PCM maintenance therapy with IMiDs. Maintenance plans should not be altered since the benefits greatly outweigh the likelihood of a SPM, however there should be a low threshold to obtain a bone marrow biopsy in PCM patients on IMiD therapy who develop unexplained cytopenias. Treatment regimens for B-ALL following PCM have varied significantly and therefore more reports are required to guide decision-making in this context.

## Data Availability

All relevant data is contained within the manuscript. The raw data is not made available due to confidentiality.

## Abbreviations

AHSCT: Autologous hematopoietic stem cell transplant
AML: Acute myeloid leukemia
B-ALL: B-Cell acute lymphoblastic leukemia
DVd: Liposomal doxorubicin, vincristine, and dexamethasone
FISH: Fluorescent in situ hybridization
HL: Hodgkin’s lymphoma
IMiD: Immunomodulatory imide drug
IMWG: International Myeloma Working Group
InO: Inotuzumab ozogamicin
M protein: Monoclonal protein
MDS: Myelodysplastic syndrome
MGUS: Monoclonoal gammopathy of undetermined significance
NCI: National Cancer Institute
NHL: Non-Hodgkin’s lymphoma
PCM: Plasma cell myeloma
SEER: Surveillance. Epidemiology. and End Results
SPEP: Serum protein electrophoresis
SPM: Second primary malignancy
VAD: Vincristine, doxorubicin, dexamethasone
WBC: White blood cell
